# Evaluating the effect of Japan’s 2004 postgraduate training programme on the spatial distribution of physicians

**DOI:** 10.1186/1478-4491-13-5

**Published:** 2015-01-24

**Authors:** Rie Sakai, Hiroshi Tamura, Rei Goto, Ichiro Kawachi

**Affiliations:** Department of Social and Behavioural Sciences, Harvard School of Public Health, 677 Huntington Avenue, Boston, MA 02215 USA; Department of Medical Education, Juntendo University School of Medicine, Hongo 2-1-1 Bunkyo-ku, Tokyo, Japan; Department of Paediatrics and Adolescent Medicine, Juntendo University School of Medicine, Hongo 2-1-1 Bunkyo-ku, Tokyo, Japan; Division of Medical Information Technology and Administration Planning, Kyoto University Hospital, Sakyo-ku, Kyoto City, Kyoto, 606-8507 Japan; Department of Ophthalmology and Visual Sciences, Kyoto University Graduate School of Medicine, Sakyo-ku, Kyoto City, Kyoto, 606-8507 Japan; Hakubi Centre of Advanced Research, Kyoto University, Sakyo-ku, Kyoto City, Kyoto, 606-8501 Japan; Graduate School of Economics, Kyoto University, Yoshida, Sakyo-ku, Kyoto, Japan

**Keywords:** Human resources, Physician distribution, Postgraduate medical training programme, Japan

## Abstract

**Background:**

In 2004, the Japanese government permitted medical graduates for the first time to choose their training location directly through a national matching system. While the reform has had a major impact on physicians’ placement, research on the impact of the new system on physician distribution in Japan has been limited. In this study, we sought to examine the determinants of physicians’ practice location choice, as well as factors influencing their geographic distribution before and after the launch of Japan’s 2004 postgraduate medical training programme.

**Methods:**

We analyzed secondary data. The dependent variable was the change in physician supply at the secondary tier of medical care in Japan, a level which is roughly comparable to a Hospital Service Area in the US. Physicians were categorized into two groups according to the institutions where they practiced; specifically, hospitals and clinics. We considered the following predictors of physician supply: ratio of physicians per 1,000 population (physician density), age-adjusted mortality, as well as measures of residential quality. Ordinary least-squares regression models were used to estimate the associations. A coefficient equality test was performed to examine differences in predictors before and after 2004.

**Results:**

Baseline physician density showed a positive association with the change in physician supply after the launch of the 2004 programme (*P-*value < .001), whereas no such effect was found before 2004. Urban locations were inversely associated with the change in physician supply before 2004 (*P-*value = .026), whereas a positive association was found after 2004 (*P-*value < .001). Urban location and area-level socioeconomic status were positively correlated with the change in hospital physician supply after 2004 (*P-*values < .001 for urban centre, and .025 for area-level socioeconomic status), even though in the period prior to the 2004 training scheme, urban location was inversely associated with the change in physician supply (*P-*value = .015) and area-level socioeconomic status was not correlated.

**Conclusion:**

Following the introduction of the 2004 postgraduate training programme, physicians in Japan were more likely to move to areas with already high physician density and urban locations. These changes worsened regional inequality in physician supply, particularly hospital doctors.

## Background

Optimizing physician supply and distribution are vital ingredients of sustaining equitable access to essential health services. However, there may be a tension between expanding access to physician supply versus improving the quality of their training, as our case study from Japan will illustrate.

In 2004, the Japanese Ministry of Health, Labour and Welfare (MHLW) sought to improve the quality of medical residency training nationwide by instituting a new postgraduate medical education (PGME) programme [1]. Prior to 2004, medical graduates commenced their specialty training immediately after graduating from medical school. Since medical graduates ended up being trained only in their chosen specialty, they had little or no experience in other fields. Since 2004, a 2-year general residency has been required of all medical graduates, ensuring that residents rotate through different specialties to gain hands-on experience at teaching hospitals designated by MHLW. These hospitals would include both university-affiliated and non-university-affiliated hospitals, encompassing both public and private institutions [2]. After 2004, new physicians commenced their specialty training following the mandatory 2-year residency training programme.

Before 2004, a majority of new graduates entered their specialty training programme at the university hospital associated with the medical school where they graduated. In those times, physicians were dispatched to non-university hospitals by the department of their specialization in the university hospital, and individual choice was not considered.

As part of the 2004 reforms, the Ministry permitted medical graduates to choose their preferred residency location directly through a national matching system for the first time [2]. Then, medical graduates tended to select independent urban hospitals not affiliated with a university for their training rather than university hospitals [3, 4]. Even though the matching system only applied to the 2-year residency training programme, young physicians now had the freedom to choose a residency location that could also provide them the specialty training, in terms of targeted mentors and professional recognition, when the residency was completed. As a result, young physicians tended to stay at the same location and continue their specialty training after the residency training. Subsequently, the number of incoming physicians at university hospitals decreased. Medical specialty departments in university (teaching) hospitals normally dispatch physicians at all levels to other affiliated hospitals. Because the number of physicians at university hospitals has decreased, university hospitals find it increasingly difficult to send physicians to affiliated hospitals, which are often located in rural and underserved areas [5]. Furthermore, physicians working in rural areas return to university hospitals to fill the void left by the lack of new medical graduates. The Japan Medical Association Research Institute reported that almost 60% of all university hospitals have reduced the dispatch of physicians to other medical institutions since the launch of the new training programme [6].

To determine if the 2004 reform had the unintended consequence of impacting physician supply and distribution, we performed an ecological analysis using nationally available data.

In a previous study, we examined the impact of the 2004 reform on the supply of paediatricians in Japan [7]. Our previous study suggested that the 2004 medical residency scheme had the unintended consequence of making it easier for new graduates to choose residency training location, exacerbating regional inequalities in paediatrician supply. A limitation of our previous study was that we ignored the distinction between hospital-based clinicians and smaller-clinic-based physicians (that is primary care clinics with fewer than 20 beds). The 2004 reforms affected resident training programmes that take place primarily in training hospitals designated by the MHLW, as opposed to physicians’ practice locations in small clinics. Therefore, the spatial distribution for clinic-based physicians would not be expected to be affected by the new scheme.

Indeed Toyabe [8] analyzed the time trends in the number and distribution of physicians between 1996 and 2006 and showed that the increasing inequality in geographic distribution of hospital-based clinicians was markedly worse compared to clinic-based physicians; moreover, the number of physicians working at hospitals increased over time in urban areas but not in areas with low population density.

In this study, we sought to disaggregate the hospital clinician trends from clinic-based physicians. We are interested in 2 questions: first, what are the principal drivers of physicians’ practice location choice, and second, what factors affected physician distribution before and after the launch of the new programme in 2004?

### Data and methods

#### Unit of analysis

We conducted an ecological analysis of physician supply and distribution. The Japanese medical system is organized into three tiers of administration: national, prefectural and municipal. Japan has 47 prefectures, and during the study period underwent administrative re-organization through a large-scale merging of municipalities which decreased the total number of municipalities from 3,232 to 1,750 between 1998 and 2010. Data are adjusted to reflect new municipal boundaries by merging former smaller municipalities into larger ones. The main unit of analysis used for our study is the ‘secondary tier of medical care’ (STM), typically comprising several municipalities. Each prefecture encompasses four to ten STMs based on medical resources, transportation, and geography, and are roughly comparable to Hospital Service Areas in the US. STMs are considered independent administrative areas from a health service perspective, and are less prone to local spillovers compared to municipalities or counties (typically used in other studies) [8–14]. The total number of STMs did not change significantly during the study period, which was between 1998 and 2010, although borders were redrawn. The STM boundaries from 2010 (n = 346) were used in this study.

### Data

#### Dependent variable

Our main dependent variables of interest are differences in numbers of physicians between the 2 4-year time periods: 1998 to 2002, which represents the period before the 2004 programme started (*pre*-*period*), and 2006 to 2010, which represents the period after the introduction of the 2004 programme (*post*-*period*). That is to say, the results can be interpreted as such: when the estimates are negative, the predictors are related to decreases in the number of physicians. When the estimates are positive, the predictors are related to increases in the number of physicians.

Only physicians who mainly worked as health care service providers are included, while physicians who were engaged in basic research or government service were excluded from the analysis. Physicians were categorized into two groups according to the institutions where they practiced: hospitals versus clinics.

#### Independent variables

The two main predictors of interest for the change in physician supply analysis were: measures of need and measures of residential quality, as generally highlighted in the literature on physicians’ practice location choice [15–18]. The 2 primary markers of need that we used were: physicians per 1,000 population (physician density), and age-adjusted mortality rates at the beginning of the 2 periods.

We considered urban/rural status as an indicator of residential quality. Municipalities were divided into five categories, based on the metropolitan area code defined by the Ministry of Internal Affairs and Communications: 1) central cities of major metropolitan areas, 2) central cities of metropolitan areas, 3) surrounding municipalities of central cities of major metropolitan areas, 4) surrounding municipalities of central cities of metropolitan areas, and 5) other municipalities. This classification is revised, based on the results of the national census in Japan, which is conducted every 5 years.

The classification from 2000 and 2005 (aligned to census years) was used to characterize the *pre*-*period* (1998 to 2002) and *post*-*period* (2006 to 2010), respectively. In this study, the metropolitan and the major metropolitan areas were combined into a single category, since there were only 5 central cities among 1,750 municipalities in 2000 and 6 in 2005. This resulted in 3 basic groups: 1) STMs that included central cities for major metropolitan areas and metropolitan areas, defined as urban centres (n = 26 in *pre*-*period* and n = 28 in *post*-*period*); 2) STMs that include surrounding municipalities of central cities of the metropolitan areas and the major metropolitan areas, which are defined as suburban areas (n = 127 in *pre*-*period* and n = 130 in *post*-*period*); and 3) others, which are defined as rural (n = 193 in *pre*-*period* and n = 188 in *post*-*period*). ‘Rural’ is used as a reference group in the models. Because no standard definition of the term ‘rural’ exists [19–22], we checked the robustness of the model with respect to alternative urban/rural measures. Previous studies have employed one of the following definitions [19]: 1) metropolitan statistical area [10, 21], which is comparable to metropolitan area codes in Japan; 2) population size [12, 19, 21]; and 3) population density [19, 23]. We employed population density as an alternative way to define urban/rural status. Under this alternative definition, the STMs with a population density of more than 1,000/km^2^ are defined as urban (n = 68 in *pre*-*period* and n = 67 in *post*-*period*), and the remaining are defined as rural (n = 278 in *pre*-*period* and n = 279 in *post*-*period*).

As an additional measure of local residential quality, we also included a composite area-based index of socioeconomic status (SES composite index), which was created from indicators representing education, occupation, and income. The index was derived from a factor analysis of the percentage of the local population with a college-level education, the percentage of white-collar workers, the unemployment rate, and *per capita* income. Factor scores, formulated by a principal component analysis with varimax rotation, were used to construct a composite index to represent each aspect of SES for the study units [24].

Table 1 describes each of the variables selected in the models. In addition to measures of local need and residential quality, we considered measures of professional interactions because the published literature has suggested that professional interactions were important factors that affect physicians’ decisions regarding their practice locations [15–18]. Meteorological data on average temperature and humidity were available only at the prefecture level.

**Table 1:** **Variables selected in the models**

Variables	Explanation
**Measures of needs**	
Age-adjusted mortality	
Physician density	The number of physicians who work for any medical facilities per 1,000 population
Hospital physician density	The number of physicians who work for hospitals per 1,000 population
Clinic physician density	The number of physicians who work for clinics per 1,000 population
**Measures of residential quality**	
Urban/rural status	The metropolitan area code defined by the Ministry of Internal Affairs and Communications
1) urban centres, 2) suburban areas,	
3) rural areas	
*Per capita* income	
Percent of the population	As a proxy for educational level in the community
with a university-level education	
Unemployment rate	The number of unemployed individuals per the number of all individuals currently in the labour force (workforce)
Percent of white-collar workers	The number of professionals, technical workers, managers, and administrators per number of workforce
Primary school students per number	As a proxy for children’s educational opportunities
of primary schools	
Crime rate	The number of crimes per total population as a proxy for neighbourhood safety
Temperature	As a proxy for climate discomfort. The discomfort index was calculated by using temperature and humidity and used in the model.
Humidity	
**Other factors**	
Total population	
Hospital beds per 1,000 population	
The presence or absence of	As a proxy for continuing education
medical schools	

### Data sources

We obtained data from multiple sources. Data for physician totals were obtained from the Survey of Physicians, Dentists, and Pharmacologists [25], which is conducted every 2 years by the MHLW. All licensed physicians are expected to complete this survey and register their working addresses and specialties under the Medical Practitioners Law [26]. The local population by age group of 5-year-olds was obtained from the Basic Resident Registers [27] and was used to calculate physician-to-population ratios.

Factors previously shown to be associated with physician supply [15–18] were obtained from publicly-available secondary data. Numbers of deaths by age group of 5-year-olds were obtained from the vital registration system [28–31]. The oldest data yielded mortality dates back to 1999, so the age-adjusted mortality of 1999 was applied in the analysis of the period 1998 to 2002. The age-adjusted mortality from 2006 was used in the analysis of the period 2006 to 2010. For the calculation of age-adjusted mortality, direct age-standardization was applied using the 1985 model population of Japan as the standard.

The data for the following five variables were obtained from the *Regional Statistics by Municipalities*, which were produced by the Ministry of Internal Affairs and Communications (MIAC) [32]: 1) *per capita* income; 2) number of hospital beds; 3) number of primary schools; 4) number of primary school students; and 5) crime rates, defined as number of crimes per 1,000 population. The data for the following 4 variables were obtained from the 2000 and 2005 Japanese Census [33–35]: 1) metropolitan area codes; 2) percentage of the population with a college-level education; 3) unemployment rate; and 4) percentage of white-collar workers. Unemployment rates and the percentage of white-collar workers were calculated using the mean of 1995 and 2000 data and applied to the time period 1998 to 2002. Corresponding data from 2005 were applied to analysis of the time period 2006 to 2010. The percentage of the population with a college-level education is only collected every 10 years. The data from 1990 were no longer publicly available. Therefore, data from 2000 were applied to the time period 1998 to2002. The mean of 2000 and 2010 data were applied to the time period 2006 to 2010.

To assess average climate (as a potential factor in physician location preference), the discomfort index, developed by the US Weather Bureau (currently the National Weather Service) and widely used in previous studies [36, 37], was calculated by using temperature and humidity. Temperature and humidity could only be obtained at the prefecture level; data for the model were retrieved from *Regional Statistics by Prefectures*, which was produced by MIAC [38].

### Statistical analysis

Descriptive statistics of all continuous variables were presented as means with standard deviations and 95% confidence intervals (CIs) for the periods 1998 to 2002 (*pre*-*period*) and 2006 to 2010 (*post*-*period*). Mean equality tests were performed to examine the statistical significance of the observed differences.

Ordinary least-squares regression models were used to analyze changes in STM-level physician supply in the *pre*- and *post*-*period*. For both periods, changes in physician supply were evaluated as a function of STM-level baseline factors, which were defined as 1998 conditions for the *pre*-*period* and 2006 conditions for the *post*-*period*. A test of coefficient equality in regressions was performed to examine significant differences in coefficients between the *pre*- and the *post*-*period*.

To examine if the main predictors of interest had meaningful effects on the change in physician supply, after accounting for the effects of the control variables, likelihood ratio tests (LRTs) were performed to compare the models that only included control variables with the ones that included each main predictor of interest or all of these predictors together with the control variables. By doing these LRTs, we can check whether each and all of the predictors have a significant contribution to the explanation of changes in physician supply in the *pre-* or *post-period*, that could not already be accounted for by the control variables.

As a robustness check, the under-5-year-old population and the over-65-year-old population were used to calculate physician density, because these age groups tend towards greater demand for medical services [15].

A two-tailed *P*-value of less than .05 was considered statistically significant. All analyses were performed using SAS 9.2 software (SAS Institute, Inc., Cary, NC, USA). This study does not directly involve the use of human subjects, nor their identifiable data, but aggregated and de-identified data that was released by the Japanese government and is publicly-available. The study was consistent with the Declaration of Helsinki.

## Results

Table 2 shows the aggregate level change in physician supply. Both the absolute number of physicians and the physician-to-population ratio increased in both the *pre*- and *post*-*periods*.

**Table 2:** **The aggregate level change in physician supply at the national level**

	1998	2002	4-year relative change (2002 to 1998)/1998	2006	2010	4-year relative change (2010 to 2006)/2006
Number of all physicians (A = B + C)	236,933	249,574	5.34%	263,540	280,431	6.41%
Number of physicians working at the hospitals (B)	153,100	159,131	3.94%	168,327	180,966	7.51%
Number of physicians working at the clinics (C)	83,833	90,443	7.88%	95,213	99,465	4.47%
Population (millions) (D)	125.57	126.46	0.71%	127.06	127.06	0.00%
Physician density^a^ (A/D)	1.89	1.97	4.60%	2.07	2.21	6.41%
Hospital physician density^b^ (B/D)	1.22	1.26	3.21%	1.32	1.42	7.51%
Clinic physician density^c^ (C/D)	0.67	0.72	7.13%	0.75	0.78	4.46%

^a^Number of all physicians per 1,000 population.

^b^Number of physicians working at hospitals per 1,000 population.

^c^Number of physicians working at health care facilities per 1,000 population.

Table 3 shows descriptive statistics of dependent variables, which are differences in numbers of physicians between both 4-year time periods: 1998 to 2002 for the *pre*-*period*, and 2006 to 2010 for the *post*-*period*, and continuous independent variables for the *pre*- and *post*-*periods*, along with the *P*-value resulting from testing the Equality of Means. Overall physician density increased (*P*-values for overall physician density was .001, for hospital physician density was .011, and for clinic physician density was < .0001). Table 4 shows descriptive statistics of categorical independent variables for the *pre*- and *post*-*periods*. The number of urban, suburban, and rural centres changed slightly between the *pre-* and *post-period*, and the number of medical schools did not change during the study period.

**Table 3:** **Descriptive statistics of all dependent and continuous independent variables**, **the secondary tier of medical care as a unit of analysis** (**n** = **346**) **The numbers after each variable name indicate the years used in the analyses**

	1998 to 2002			2006 to 2010			
	Mean	SD ^a^	95% CIs ^b^	Mean	SD ^a^	95% CIs ^b^	***P -value*** ^***c***^
Changes in number of all physicians	36.53	62.10	[29.97 to 43.1]	48.82	120.76	[36.05 to 61.59]	.093
Changes in number of physicians working at the hospital	17.43	38.98	[13.31 to 21.55]	36.53	93.92	[26.6 to 46.46]	< .001
Changes in number of physicians working at the clinics	19.10	35.83	[15.32 to 22.89]	12.29	35.07	[8.58 to 16.00]	.012
Number of all physicians 1998/2006	703.00	1,000.90	[628.3 to 777.7]	786.10	1,144.90	[700.6 to 871.5]	.151
Number of physicians working at the hospital 1998/2006	451.20	670.90	[401.1 to 501.3]	504.80	770.70	[447.2 to 562.3]	.168
Number of physicians working at the clinic 1998/2006	251.80	346.80	[226.0 to 277.7]	281.30	390.70	[252.2 to 310.5]	.138
Age-adjusted mortality 1999/2006	1.15	0.07	[1.15 to 1.16]	0.99	0.06	[0.99 to 1.00]	< .001
Physician density^d^ 1998/2006	1.70	0.80	[1.64 to 1.76]	1.85	0.85	[1.78 to 1.91]	.001
Hospital physician density^e^ 1998/2006	1.07	0.63	[1.03 to 1.12]	1.16	0.68	[1.11 to 1.12]	.011
Clinic physician density^f^ 1998/2006	0.63	0.22	[0.61 to 0.64]	0.68	0.23	[0.67 to 0.70]	< .001
Total population (thousands) 1998/2006	364.20	399.50	[334.40 to 394.00]	367.20	417.70	[336.00 to 398.40]	.891
*Per capita* income (thousands)^g^ 1998/2006	11.75	3.31	[11.51 to 12]	11.36	3.15	[11.12 to 11.59]	.023
Percent of the population with a college-level education 2000/((2000 + 2010)/2)^h^	10.81	5.25	[10.26 to 11.37]	11.92	5.39	[11.35 to 12.49]	.006
Unemployment rate ((1995 + 2000)/2)^i^/2005	4.06	1.19	[3.93 to 4.18]	5.78	1.50	[5.63 to 5.94]	< .001
Percent of white-collar workers ((1995 + 2000)/2)^i^/2005	14.42	2.40	[14.17 to 14.68]	13.96	2.25	[13.73 to 14.2]	.010
Socioeconomic status (SES) composite index^j^	-0.02	1.02	[-0.13 to 0.09]	0.02	0.98	[-0.08 to 0.12]	.593
Number of primary students/school 1998/2006	281.20	133.00	[271.3 to 291.1]	274.00	138.80	[263.7 to 284.4]	.325
Crime rate 1998/2006	1.47	0.73	[1.42 to 1.52]	1.10	0.53	[1.06 to 1.14]	< .001
Temperature (°C) 1998/2006	15.82	2.53	[15.3 to 16.34]	15.59	2.36	[15.10 to 16.07]	.517
Humidity (%) 1998/2006	70.28	4.70	[69.31 to 71.25]	69.44	4.36	[68.54 to 70.33]	.205
Discomfort index^k^	60.05	3.87	[59.25 to 60.84]	59.68	3.58	[58.94 to 60.41]	.500
Hospital beds per 1,000 population 1998/2006	13.89	4.75	[13.54 to 14.24]	13.89	4.62	[13.55 to 14.24]	.990

^a^Standard deviation.

^b^Confidence intervals.

^c^ *P*-value of mean equality test.

^d^Number of all physicians per 1,000 population.

^e^Number of physicians working at hospitals per 1,000 population.

^f^Number of physicians working at health care facilities per 1,000 population.

^g^Japanese yen was converted into US$ using the rate that applied in March 2013 of approximately 95 Japanese yen per US$.

^h^The percent of college-level education is only collected every 10 years. Data from 2000 were applied to the time period 1998 to 2002, and the mean of 2000 and 2010 data were applied to the time period 2006 to 2010.

^i^Unemployment rates and the percentage of white-collar workers were calculated using the mean of 1995 and 2000 data and applied to the time period 1998 to2002.

^j^A composite index of socioeconomic indicators created from the percent of the population with a college-level education, percent of white-collar workers, the unemployment rate, and *per capita* income.

^k^Calculated by using temperature and humidity.

**Table 4:** **Descriptive statistics of categorical independent variables**, **the secondary tier of medical care as a unit of analysis** (**n** = **346**)

	1998 to 2002		2006 to 2010		***P-value***
	n	(%)	n	(%)	
Urban centre	26	(7.5)	28	(8.1)	0.677
Suburban	127	(36.7)	130	(37.6)	
Rural	193	(55.8)	188	(54.3)	
STM^a^s with at least one medical school	65	(18.79)	65	(18.79)	1.000

^a^Secondary tier of medical care.

Table 5 presents the results of our main predictors of interest from the multivariate ordinary least-squares regression models controlled by all other factors shown in Table 1 for all physicians. While the change in physician supply was not associated with baseline physician density in the *pre*-*period*, it was positively associated with baseline physician density in the *post*-*period* (*P*-value < .0001). In other words, in the post-2004 period, areas with already high physician supply attracted even more physicians. After adjustment for all other variables, we estimated that each unit increase in physician density (1 physician per 1,000 population) in 2006 was associated with an increase in the number of physicians of 64.80 in 2010. While the change in physician supply was inversely associated with urban centres in the *pre*-*period* (*P*-value .026), it was positively associated with urban centres in the *post*-*period* (*P*-value < .0001). On average, we estimated that urban centres lost 26.71 physicians from 1998 to 2002 and gained 68.81 from 2006 to 2010, compared to rural areas after adjustment for all other variables.

**Table 5:** **Results of multivariate ordinary least**-**squares regression models for all physicians** ^**a**^

	1998 to 2002				2006 to 2010				***P-value***of coefficient equality test
Main predictors of interest	Estimate coefficient	SE ^b^	95% CIs ^c^	***P- value***	Estimate coefficient	SE ^b^	95% CIs ^c^	***P- value***	
**Measure of public health need**									
Age-adjusted mortality	-97.07	33.42	[-162.58 to -31.56]	.004	108.90	61.86	[-12.35 to 230.15]	.078	.004
Physician density^d^	-5.55	4.32	[-14.02 to 2.92]	.199	64.80	6.79	[51.5 to 78.11]	< .001	< .001
**Measure of residential quality**									
Urban centre	-26.71	11.99	[-50.21 to -3.21]	.026	68.81	17.05	[35.4 to 102.22]	< .001	< .001
Suburban	-5.86	5.33	[-16.31 to 4.59]	.272	-5.04	8.05	[-20.83 to 10.74]	.531	.933
Rural area	Reference			Reference					
SES composite index^e^	6.96	3.77	[-0.43 to 14.35]	.065	18.64	5.72	[7.42 to 29.86]	.001	.089

^a^The models included the control variables: total population, number of primary school students per number of primary schools, crime rate, discomfort index calculated by temperature and humidity, hospital beds per 1,000 population, and the presence or absence of medical schools.

^b^Standard error.

^c^Confidence intervals.

^d^Ratio of number of physicians to 1,000 population.

^e^Socioeconomic status (SES) composite index was created from the percent of the population with a college-level education, percent of white-collar workers, the unemployment rate, and *per capita* income.

Table 6 shows the results from the LRT for all physicians. Physician density, urban/rural status, and the SES composite index did not have meaningful effects on the change in physician supply in the *pre*-*period*, while they had significant effects in the *post*- *period*.

**Table 6:** **Results from likelihood ratio test (LRT) for all physicians**

	R squared	LR statistic ^a^	DF ^b^	***P-value***	R squared	LR statistic ^a^	DF ^b^	***P-value***
Model only with control variables (#1)^c^	0.625				0.648			
#1 + Age-adjusted mortality^d^	0.636	10.81	1	.001	0.652	4.75	1	.029
#1 + Physician density^e^	0.624	0.15	1	.697	0.764	139.67	1	< .001
#1 + urban/rural status^f^	0.627	4.24	2	.120	0.679	34.05	2	< .001
#1 + SES composite index^g^	0.627	3.14	1	.077	0.701	57.18	1	< .001
Full model^h^	0.640	18.95	5	.002	0.783	172.10	5	< .001

^a^The likelihood ratio test statistic.

^b^Degree of freedom.

^c^The models included only control variables, which are total population, number of primary school students per number of primary schools, crime rate, discomfort index calculated by temperature and humidity, hospital beds per 1,000 population, and the presence or absence of medical schools.

^d^The models included control variables and age-adjusted mortality.

^e^The models included control variables and ratio of number of physicians to 1,000 population.

^f^The models included control variables and urban centre and suburban.

^g^The models included control variables and socioeconomic status (SES) composite index, which was created from the percent of the population with a college-level education, percent of white-collar workers, the unemployment rate, and *per capita* income.

^h^The models included all variables.

Table 7 shows the results from the multivariate ordinary least-squares regression analyses for hospital-based clinicians. While the change in hospital physician supply was inversely associated with baseline hospital physician density in the *pre*-*period* (*P*-value = .047), it was positively associated with baseline hospital physician density in the *post*-*period* (*P*-value < .001). Urban centres and the SES composite index showed statistically-positive associations with the change in hospital physician supply in the *post*-*period* (*P*-values < .001 for urban centres, and .025 for SES c index), whereas the change in hospital physician supply was inversely associated with urban centres and no such effects of SES composite index during the *pre*-*period* (*P*-value = .*015 for urban centres and .483 for SES composite index*). The coefficient equality test showed significant differences in coefficients between the *pre*-*period* and *post*-*period* both for urban centres (*P*-value < .0001) and the SES composite index (*P*-value = .026).

**Table 7:** **Results of multivariate ordinary least**-**squares regression models for physicians working at the hospitals** ^**a**^

	1998 to 2002				2006 to2010				***P***-***value***of coefficient equality test
Main predictors of interest	Estimated coefficient	SE ^b^	95% CIs ^c^	***P*** ***-*** ***value***	Estimated coefficient	SE ^b^	95% CIs ^c^	***P***-***value***	
**Measure of public health need**									
Age-adjusted mortality	-67.61	29.87	[-126.16 to -9.06]	.024	54.76	50.47	[-44.16 to 153.69]	.278	.038
Hospital physician density^d^	-10.96	5.52	[-21.77 to -0.15]	.047	57.87	7.62	[42.92 to 72.81]	< .001	< .001
Clinic physician density^e^	7.22	12.70	[-17.67 to 32.1]	.570	33.85	16.92	[0.68 to 67.01]	.046	.209
**Measure of residential quality**									
Urban centre	-25.95	10.70	[-46.92 to -4.98]	.015	51.99	13.96	[24.62 to 79.36]	< .001	< .001
Suburban	-0.72	4.78	[-10.08 to 8.64]	.880	-5.11	6.58	[-18.02 to 7.79]	.437	.590
Rural area	Reference				Reference				
SES composite index^f^	-2.32	3.30	[-8.79 to 4.16]	.483	10.50	4.67	[1.35 to 19.65]	.025	.026

^a^The models included the control variables: total population, number of primary school students per number of primary schools, crime rate, discomfort index calculated by temperature and humidity, hospital beds per 1,000 population, and the presence or absence of medical schools.

^b^Standard error.

^c^Confidence intervals.

^d^Ratio of number of physicians working at the hospitals to population.

^e^Ratio of number of physicians working at the clinics to population.

^f^Socioeconomic status (SES) composite index was created from the percent of the population with a college-level education, percent of white-collar workers, the unemployment rate, and *per capita* income.

Table 8 shows the results from the LRT for physicians working at the hospitals. Hospital physician density and urban/rural status had meaningful effects on the change in hospital physician supply both in the *pre*- and *post*-*periods*. However, both estimates were negative in the *pre-period*, and positive in the *post-period*. Therefore, the effects of those variables were meaningful in both periods in an opposite direction. SES composite index had significant effect in the *post*-*period*, while there was no such effect in the *pre*-*period*.

**Table 8:** **Results from likelihood ratio test (LRT) for physicians working at the hospitals**

	R squared	LR statistic ^a^	DF ^b^	***P- value***	R squared	LR statistic ^a^	DF ^b^	***P- value***
Model only with control variables (#1)^c^	0.274				0.632			
#1 + Age-adjusted mortality^d^	0.279	3.20	1	.074	0.637	5.64	1	.018
#1 + hospital physician density^e^	0.291	9.21	1	.002	0.742	123.55	1	< .001
#1 + clinic physician density^f^	0.279	3.07	1	.080	0.689	59.87	1	< .001
#1 + urban/rural status^g^	0.291	10.29	2	.006	0.662	31.85	2	< .001
#1 + SES composite index^h^	0.274	0.90	1	.342	0.676	45.05	1	< .001
Full model^i^	0.310	23.39	6	.001	0.761	156.11	6	< .001

^a^The likelihood ratio test statistic.

^b^Degree of freedom.

^c^The models included only control variables, which are total population, number of primary school students per number of primary schools, crime rate, discomfort index calculated by temperature and humidity, hospital beds per 1,000 population, and the presence or absence of medical schools.

^d^The models included control variables and age-adjusted mortality.

^e^The models included control variables and ratio of number of physicians working at the hospitals to population.

^f^The models included control variables and ratio of number of physicians working at the clinics to population.

^g^The models included control variables and urban centre and suburban.

^h^The models included control variables and socioeconomic status (SES) composite index, which was created from the percent of the population with a college-level education, percent of white-collar workers, the unemployment rate, and *per capita* income.

^i^The models included all variables.

Table 9 shows the results from the multivariate ordinary least-squares regression analyses for clinic-based physicians, including clinic owners. The change in clinic physician supply was positively associated with baseline hospital physician density both in the *pre*- and *post*-*period* (*P*-values = .*001* for *pre*-*period* and < .001 for *post*-*period*). The coefficient equality test suggested that there were significant differences in coefficients between the *pre*-*period* and *post*-*period* for hospital physician density (*P*-value of coefficient equality test = .006). The change in clinic physician supply was inversely associated with baseline clinic physician density both in the *pre*- and *post*-*period* (*P-*values = .013 for *pre*-*period*, and .022 for *post*-*period*) and there were no significant changes in the estimated impact of these factors between the 2 periods. Urban centres showed statistically positive associations with the change in clinic physician supply in the *post*-*period* (*P-*value = .*003*). Although it did not show statistically positive associations with the change in clinic physician supply in the *pre*-*period*, the coefficient equality test showed there were no significant differences in coefficients between the *pre*-*period* and *post*-*period* for urban centres (*P-*-value = .050). The SES composite index showed statistically positive associations with the change in clinic physician supply both in the *pre*- and *post*-*period* (both *P-*values < .001).

**Table 9:** **Results of multivariate ordinary least**-**squares regression models for physicians working at the clinics** ^**a**^

	1998 to 2002				2006 to 2010				***P-*** ***value***of coefficient equality test
Main predictors of interest	Estimated coefficient	SE ^b^	95% CIs ^c^	***P-*** ***value***	Estimated coefficient	SE ^b^	95% CIs ^c^	***P-*** ***value***	
**Measure of public health need**									
Age-adjusted mortality	-30.98	16.38	[-63.08 to 1.12]	.059	49.23	25.59	[-0.93 to 99.39]	.0544	.009
Hospital physician density^d^	9.90	3.02	[3.98 to 15.83]	.001	23.53	3.87	[15.95 to 31.1]	< .001	.006
Clinic physician density^e^	-17.27	6.96	[-30.92 to -3.63]	.013	-19.64	8.58	[-36.46 to -2.83]	.022	.830
**Measure of residential quality**									
Urban centre	2.90	5.87	[-8.6 to 14.39]	.622	21.00	7.08	[7.12 to 34.88]	.003	.050
Suburban	-4.84	2.62	[-9.97 to 0.29]	.064	1.50	3.34	[-5.05 to 8.04]	.654	.136
Rural area	Reference				Reference				
SES composite index^f^	7.55	1.81	[4.01 to 11.1]	<.001	8.57	2.37	[3.93 to 13.21]	< .001	.734

^a^The models included the control variables: total population, number of primary school students per number of primary schools, crime rate, discomfort index calculated by temperature and humidity, hospital beds per 1,000 population, and the presence or absence of medical schools.

^b^Standard error.

^c^Confidence intervals.

^d^Ratio of number of physicians working at the hospitals to population.

^e^Ratio of number of physicians working at the clinics to population.

^f^Socioeconomic status (SES) composite index was created from the percent of the population with a college-level education, percent of white-collar workers, the unemployment rate, and *per capita* income.

Table 10 shows the results from the LRT for physicians working at the clinics. Urban–rural status and SES composite index, which had meaningful effects on the change in hospital physician supply between *pre*- and *post-periods*, also had meaningful effects on the clinic physician supply both in *pre*- and *post*-*periods*.

**Table 10:** **Results from likelihood ratio test (LRT) for physicians working at the clinics**

	R squared	LR statistic ^a^	DF ^b^	***P- value***	R squared	LR statistic ^a^	DF ^b^	***P- value***
Model only with control variables (#1)^c^	0.709				0.449			
#1 + Age-adjusted mortality^d^	0.718	11.37	1	.001	0.448	0.62	1	.429
#1 + hospital physician density^e^	0.728	24.64	1	< .001	0.532	57.54	1	< .001
#1 + clinic physician density^f^	0.709	1.42	1	.233	0.459	7.79	1	.005
#1 + urban/rural status^g^	0.714	8.65	2	.013	0.467	13.87	2	< .001
#1 + SES c index^h^	0.730	27.46	1	< .001	0.500	34.66	1	< .001
Full model^i^	0.747	54.20	6	< .001	0.560	83.92	6	< .001

^a^The likelihood ratio test statistic.

^b^Degree of freedom.

^c^The models included only control variables, which are total population, number of primary school students per number of primary schools, crime rate, discomfort index calculated by temperature and humidity, hospital beds per 1,000 population, and the presence or absence of medical schools.

^d^The models included control variables and age-adjusted mortality.

^e^The models included control variables and ratio of number of physicians working at the hospitals to population.

^f^he models included control variables and ratio of number of physicians working at the clinics to population.

^g^The models included control variables and urban centre and suburban.

^h^The models included control variables and socioeconomic status (SES) composite index, which was created from the percent of the population with a college-level education, percent of white-collar workers, the unemployment rate, and *per capita* income.

^i^The models included all variables.

All the robustness checks showed the similar results. (Detailed results are available upon request).

## Discussion

The current study explored community-level factors that affected changes in physician supply in Japan, as well as the impact of the 2004 national training programme on physician supply. We found that the determinants of the change in physician supply differed before and after the launch of the 2004 PGME programme for the following two reasons. First, physicians tended to move to places with higher physician density after the launch of the programme. Second, physicians tended to leave urban centres before the launch of the 2004 programme, whereas they tended to move to urban centres afterwards. The LRTs supported these phenomena, that is physician density was an important variable in the *post*-*period*, while it was not in the *pre*-*period*, and urban/rural status were important variables in *post*-*period*, while they were not in the *pre*-*period*. In short, the PGME programme - which was introduced to improve the quality of medical training - had the unintended consequence of worsening geographical inequality in physician supply. The essence of this story is that our disaggregated analysis revealed that the deterioration in physician supply to rural and lower-SES areas primarily affected hospital-based clinicians. More specifically, before the launch of the 2004 programme, physicians working at hospitals tended to go to locations with lower physician density, while the opposite trend was observed after the launch of the new programme. Second, physicians working at hospitals tended to move to urban centres and higher SES communities in the *post*-*period*, whereas no such effects were found *pre*-*period*; moreover, for physicians working at the clinics, tendencies to move to urban centres and higher SES communities were observed in both *pre*- and *post*-*periods*. The LRTs also supported these phenomena, that is hospital physician density and urban/rural status were important variables in both *pre*- and *post*-*period* for physicians working at hospitals; however, the direction of the effects were the opposite, and urban/rural status and SES composite index were important variables in both *pre*- and *post*-*periods for* physicians working at clinics.

The public perception in the Japanese media (also acknowledged by the government) is that regional inequality in physician supply worsened after the launch of the 2004 programme because many physicians - when allowed to choose freely - prefer work in urban areas [5, 39]. However, published quantitative analyses of the 2004 programme’s impact on physician distribution have previously failed to corroborate these views [11, 40–42]. The current study showed that our results are more in line with public perception.

There are several possible reasons why physicians favour areas with higher hospital physician density. First, in Japan, salaries of physicians at hospitals are not based on a fee-for-service structure [43]; therefore, there is not much sense of competition among hospital physicians. Rather, a colleague will complement the workload. Therefore, physicians working in hospitals with higher physician density tend to enjoy lower workloads. Additionally, when physicians need to admit patients, but hospitals do not have beds for the patients, physicians look for the beds in other hospitals. It is easier for physicians in the areas with more hospitals and physicians to find beds for patients. Also, Krishnan [15] noted that there is a tendency among physicians to prefer locations near their colleagues to benefit from professional interactions. The Japanese government raised the number of medical school admissions to increase the number of physicians until 2019. Available evidence indicates that only physician-rich geographic areas have been able to develop their hospital facilities and enhance their relative physician supply [44], providing more evidence of collegial preferences.

Another interesting result from the disaggregated analyses was that physicians working at the hospitals tended to move to urban centres and communities with higher SES *post*-*period*, whereas no such tendency was found *pre*-*period*. This suggests that residential quality has emerged as a driving force in hospital physicians’ location preference following the 2004 legislation. Our disaggregated analyses indicate that clinic-based physicians tended to move to urban centres and communities with higher SES both before and after the launch of the 2004 programme, and there were no significant differences between the two periods. Physicians at clinics had been able to decide their preferred practice location even before the launch of the 2004 programme, while the physicians at hospitals gained that freedom only after the launch. Our results showed that physicians will move to urban areas and areas with higher SES when the practice location choice is left to individual freedom. As revealed in our previous study [7], physicians are no different from the rest of the Japanese population in preferring to move to urban areas [45].

The critical challenge in most settings has been that of recruiting physicians to rural areas, where physician coverage is generally low and child health often significantly poorer compared to urban areas. Since available evidence demonstrates that students of rural origin are more likely to work in rural settings [46–48], some rural local governments adopted a programme, which reimburses medical school tuition for those students who agree to work in the rural areas to which they are assigned for a designated number of years after graduation. The government has started discussions to establish training courses that can help rural physicians obtain their board-certified specialist status after their mandatory training in the rural areas so that more physicians will stay in rural areas even after the mandatory periods. We believe that the results of our study will contribute to the discussion. We would also like to note that the policy to reimburse medical school tuition for those students who agree to work in the rural areas for a designated number of years was first instituted on a large scale in 2006 [46]. The first graduates covered by this policy graduated in 2012; therefore, this policy does not impact the results of our study.

We have previously shown how the 2004 PGME reforms impacted the regional distribution of paediatrician supply [7, 49]. Our current analysis extends our previous conclusion by showing that the impact of the 2004 reforms has not been limited just to the supply of paediatricians. We believe that we are able to show underlying evidence that new placement schemes should be developed to achieve more equity in access to medical care in Japan.

There are some limitations to consider in interpreting the results of this study. First, publicly-available data do not indicate whether a physician works full-time or part-time. This analysis was based on an overall headcount, which might overestimate the number of physicians. In particular, the percentage of female physicians is increasing [25] and the percentage of female physicians who work part-time is higher than that of men [50]. Second, publicly-available data do not include information on physician age or gender, although previous studies have noted the effect of gender or age on differences in physicians’ practice location choices [18, 51–53]. In reality, a majority of clinics in Japan are privately-owned, with the very few exceptions of clinics owned by the local governments in some rural areas. Therefore, it is possible that the data for physicians at clinics included physicians who do not provide clinical work, but are rather engaged in business administration. Last, some local governments have been implementing their own policies to attract physicians to specific (generally underserved) areas, and these policies could also influence physician practice location choice. Some regions use public funding to provide a better salary to physicians who work in rural areas to ensure physician supply in underserved areas [48]. Omitting this variable for this type of aid in the model would underestimate the coefficients reported here reflecting the true effect of the policy change on physician practice location choices. Therefore, the true effect would be even stronger than the estimated coefficients in the current study.

## Conclusions

After the 2004 PGME reforms, physicians tended to move to places with higher physician density, and the shift toward urban, higher-SES locations became more accentuated. These changes were primarily driven by hospital-based clinicians. Our study suggests that new placement schemes should be developed to achieve more equity in access to medical care in Japan.

## Abbreviations

CI: Confidence interval
LRTs: Likelihood ratio test
MHLW: The Ministry of Health, Labor and Welfare
MIAC: The Ministry of Internal Affairs and Communications
PGME: New postgraduate medical education
SES: Socioeconomic status
STM: Secondary tier of medical care.
